# Tumor cells with low proteasome subunit expression predict overall survival in head and neck cancer patients

**DOI:** 10.1186/1471-2407-14-152

**Published:** 2014-03-05

**Authors:** Chann Lagadec, Erina Vlashi, Sunita Bhuta, Chi Lai, Paul Mischel, Martin Werner, Michael Henke, Frank Pajonk

**Affiliations:** 1 Department of Radiation Oncology, David Geffen School of Medicine at UCLA, 10833 Le Conte Ave, Los Angeles, CA 90095, USA; 2 Department of Pathology, David Geffen School of Medicine at UCLA, 10833 Le Conte Ave, Los Angeles, CA 90095, USA; 3 Ludwig Institute for Cancer Research, San Diego Branch, 9500 Gilman Drive, La Jolla, CA 92039, USA; 4 Department of Pathology, University Hospital Freiburg, Breisacher Str. 115a, 79106 Freiburg, Germany; 5 Section Clinical Studies, Department of Radiation Oncology, University Hospital Freiburg, Robert-Koch-Strasse 3, D-79106 Freiburg, Germany; 6 Jonsson Comprehensive Cancer Center at UCLA, 10833 Le Conte Ave, Los Angeles, CA 90095, USA

**Keywords:** Cancer stem cells, Head and neck cancer, Proteasome, Radiotherapy

## Abstract

**Background:**

Experimental and clinical data suggest that solid cancers contain treatment-resistant cancer stem cells that will impair treatment efficacy. The objective of this study was to investigate if head and neck squamous cell carcinoma (HNSCC) also contain cancer stem cells that can be identified by low 26S proteasome activity and if their presence correlates to clinical outcome.

**Methods:**

Human HNSCC cells, engineered to report lack of proteasome activity based on accumulation of a fluorescent fusion protein, were separated based on high (ZsGreen-cODC^neg^) or low (ZsGreen-cODC^pos^) proteasome activity. Self-renewal capacity, tumorigenicity and radioresistance were assessed. Proteasome subunit expression was analyzed in tissue microarrays and correlated to survival and locoregional cancer control of 174 patients with HNSCC.

**Results:**

HNSCC cells with low proteasome activity showed a significantly higher self-renewal capacity and increased tumorigenicity. Irradiation enriched for ZsGreen-cODC^pos^ cells. The survival probability of 82 patients treated with definitive radio- or chemo-radiotherapy exhibiting weak, intermediate, or strong proteasome subunit expression were 21.2, 28.8 and 43.8 months (p = 0.05), respectively. Locoregional cancer control was comparably affected.

**Conclusions:**

Subpopulations of HNSCC display stem cell features that affect patients’ tumor control and survival. Evaluating cancer tissue for expression of the proteasome subunit PSMD1 may help identify patients at risk for relapse.

## Background

Radiotherapy is standard of care for advanced stage head and neck squamous cell carcinoma (HNSCC). However, despite high total radiation doses combined with aggressive chemotherapy the prognosis of these patients remains poor.

First introduced a century ago by Paget [1] the cancer stem cell hypothesis suggests that, similar to leukemia, solid cancers are organized hierarchically with a small number of cancer stem cells (CSCs) able to regrow a cancer and give rise to heterogeneous progeny, which lack these cancer stem cell traits [2]. Therefore, elimination of all CSCs from a tumor is a *sine qua non* for cancer cure. After a landmark paper by Al-Hajj and colleagues [3] that reported prospective identification of breast cancer stem cells, several follow-up studies provided strong clinical [4-6] and preclinical [7-10] evidence for the existence and relevance of cancer stem cells in breast cancer and glioma. The cancer stem cell hypothesis received further strong support from elegant animal experiments demonstrating the existence of cancer stem cells in undisturbed murine tumors of the GI system [11], brain [12] and skin [13]. We and others have reported that CSCs are in general resistant to established chemotherapeutic agents and are relatively radioresistant [14-18]. Thus, established treatment regimens should be re-evaluated based on their ability to kill CSCs. However, a prerequisite for such testing is the ability to identify CSCs.

Markers for the prospective identification of CSCs are relatively well defined for breast cancer [3,19-21] and glioma [7,9,10,21] while CSC markers for other solid cancers are still subject of ongoing research. A previous study suggested that CSCs in HNSCC could be prospectively identified using antibodies against the surface marker CD44 [22]. However, because CD44 is ubiquitously expressed in various isoforms, the value of CD44 as a CSC marker is controversially discussed [23]. In combination with ALDH1 staining and use of the side population CD44 still seems to be a useful marker for the prospective identification of CSCs in HNSCC [24].

We recently reported that lack of proteasome function and subunit expression is a feature of therapy-resistant, tumorigenic cells in breast cancer and glioma [16,21,25], therefore we hypothesized that HNSCCs could contain a similar cell population.

Here we report that HNSCC cell lines, indeed, contain a small population of radioresistant cells with high self-renewal capacity that can be prospectively identified based on their intrinsic low proteasome function. Furthermore, we demonstrate that a weak expression of the proteasome subunit PSMD1 in HNSCC cells predicts unfavorable outcome after radiotherapy.

## Methods

### Cell culture

Human UM-SCC4, UM-SCC6, UM-SCC12, UM-SCC-17B, FaDu, and Cal33 head and neck squamous carcinoma cell lines were a kind gift of Steven Wong (Department of Hematology/Oncology at UCLA) and have been previously described elsewhere [26]. ZsGreen-cODC expressing cells were obtained as described in Vlashi et al. [21]. Briefly, cells were infected with a retroviral vector coding for a fusion protein between the fluorescent protein ZsGreen and the C-terminal degron of murine ornithine decarboxylase. The latter targets ZsGreen to ubiquitin-independent degradation by the 26S proteasome, thus reporting lack of proteasome function through accumulation of ZsGreen-cODC. Infected cells were selected for five days using G418. Successful complete infection was verified using the proteasome inhibitor MG132 (Sigma, MO). All cell lines were cultured in log-growth phase in DMEM (Invitrogen, Carlsbad, CA) (supplemented with 10% fetal bovine serum and penicillin and streptomycin cocktail). All cells were grown in a humidified atmosphere at 37°C with 5% CO_2_.

### Irradiation

Cells grown as monolayer or sphere cultures were irradiated at room temperature using an experimental X-ray irradiator (Gulmay Medical Inc. Atlanta, GA) at a dose rate of 5.519 Gy/min for the time required to apply a prescribed dose. The x-ray beam was operated at 250 kV and hardened using a 4 mm Be, a 3 mm Al, and a 1.5 mm Cu filter. Corresponding controls were sham irradiated.

### Flow cytometry

We had previously shown that breast cancer stem cells could be identified *via* their low proteasome activity [16,21], which can be assessed by analyzing ZsGreen-cODC protein accumulation. Five days after radiation, cells were trypsinized and ZsGreen-cODC expression was assessed by flow cytometry. Cells were defined as “ZsGreen-cODC positive” if the fluorescence in the FL-1H channel exceeded the fluorescence level of 99.9% of the empty vector-transfected control cells.

Experiments were performed using a MACSquant Analyzer (Miltenyi Biotech, CA) and analyzed using the FloJo software package (vers. 9, Tree Star Inc., OR).

For ALDH1 staining, cells were fixed in 4% paraformaldehyde for 20 min at room temperature. Non-specific binding was blocked by incubating the fixed cells for 1 hour in PBS/1% BSA/0.1% Tween-20/10% goat serum at room temperature. Cells were then incubated with a mouse anti-ALDH-1 antibody (Abcam, Cambridge, MA) at 4°C overnight (1:100 dilution). After washing off the non-bound primary antibody, the cells were incubated with an anti-mouse-Cy5 secondary antibody (Abcam, Cambridge, MA) in blocking buffer for 2 hours at room temperature. Cells were then washed with PBS and analyzed on BD FACSAria.

### Sphere-forming assay

To assess sphere forming capacity, cells were trypsinized and plated in sphere media (DMEM-F12, 0.4% BSA (Sigma), 10 ml/500 ml B27 (Invitrogen) 5 μg/ml bovine insulin (Sigma), 4 μg/ml heparin (Sigma), 20 ng/ml fibroblast growth factor 2 (bFGF, Sigma) and 20 ng/ml epidermal growth factor (EGF, Sigma)) into 96-well ultra-low adhesion plates, ranging from 1 to 256 cells/well. Growth factors, EGF and bFGF, were added every 3 days, and the cells were allowed to form spheres for 21 days. The number of spheres formed per well was then counted and expressed as a percentage of the initial number of cells plated. Three independent experiments were performed.

### Animals

Nude (nu/nu), 6-8-week-old female mice, originally obtained from The Jackson Laboratories (Bar Harbor, ME) were re-derived, bred and maintained in a pathogen-free environment in the American Association of Laboratory Animal Care-accredited Animal Facilities of Department of Radiation Oncology, University of California (Los Angeles, CA) in accordance to all local and national guidelines for the care of animals.

### Tumor xenotransplantation

UM-SCC12-ZsGreen-cODC-negative, derived from monolayer cultures, and UM-SCC12-ZsGreen-cODC-positive cells derived from sphere cultures and sorted by fluorescence-activated cell sorting, were injected subcutaneously into the thighs and shoulders of 6-week old female Nu/Nu mice (10^5^, 10^4^, 10^3^, or 10^2^ cells per inoculum) within Matrigel (BD Biosciences). Tumor growth was assessed on a weekly basis, and the mice were sacrificed when the tumor size reached tumor diameters requiring euthanasia.

### Patients

Records and formalin fixed tissue blocks from patients with HNSCC irradiated between January 1997 and November 2002 were evaluated within prospective clinical trials [27-30] at the University Hospital Freiburg, Germany (Table 1). Patients were originally selected to investigate the prognostic significance of blood hemoglobin levels and cellular EpoR-expression on clinical outcome. This report will focus on data of 82 patients who received definitive radiotherapy [27,28] or radiochemotherapy [29,30] alone. Patients were older than 18 years and had histologically proven advanced (T3, T4, or nodal involvement) squamous-cell carcinoma of the oral cavity, oropharynx, hypopharynx, or larynx. For comparison, data from 92 additional patients with advanced HNSCC but receiving postoperative radiation within three of the above mentioned trials [27,29,30] will be given in Table 2.

**Table 1:** HNSCC, definitive radio- radiochemotherapy by PSMD1-score

**PSMD1-score**	**1 (n=29)**	**2 (n=26)**	**3 (n=27)**
Age (years) mean	59.5	62.1	59.3
Q1/Q2/Q3	54/59/65	56/61.5/69.7	55/60/63
Male (%)	96.5	84.6	92.5
Weight (kg) mean	72.7	72.0	71.7
Q1/Q2/Q3	60/67/81	61.5/66/79	64.5/72/80
Smoker (n)	17/26	19/24	20/26
Karnofski >= 70 (n)	11/13	8/8	6/7
Hemoglobin level (mg/dL) mean	12.7	13.5	13.2
Q1/Q2/Q3	11.9/12.9/14	12.8/13.7/14.1	12.4/13.5/14.4
Oral cavity n (%)	4 (13.7)	3 (11.5)	5 (18.5)
Oropharynx n (%)	13 (44.8)	9 (34.5)	9 (33.3)
Hypopharynx n (%)	10 (34.4)	11 (42.2)	8 (29.6)
Larynx n (%)	2 (6.8)	3 (11.5)	5 (18.5)
cT1 n (%)	1 (3.4)	1 (3.8)	
cT2 n (%)	2 (6.8)	3 (11.5)	4 (14.8)
cT3 n (%)	11 (37.9)	6 (23.0)	7 (25.9)
cT4 n (%)	15 (51.7)	16 (61.5)	16 (59.2)
cN0 n (%)	2 (6.8)	4 (15.3)	3 (11.1)
cN1 n (%)	6 (20.6)	1 (3.8)	1 (3.7)
cN2 n (%)	19 (65.3)	20 (76.8)	21 (57.7)
cN3 n (%)	2 (6.9)	1 (3.8)	2 (7.4)
G1 n (%)	2 (6.8)	2 (7.6)	
G2 n (%)	12 (41.3)	17 (65.3)	14 (53.8)
G3 n (%)	15 (51.7)	7 (26.9)	12 (46.1)
EpoR (C20+) (%)	19 (65.5)	18 (69.2)	22 (81.4)
RT (Gy) mean	71.1	69.7	67.7
Q1/Q2/Q3	70/70.6/72	69.9/70/70.6	70/70/70.6
RT (days)	48.1	47.5	45.0
Q1/Q2/Q3	42.5/48/52	42/45.5/51	42/45/50
treated in study A1/B2/C3/D4 (n)	1/10/14/4	3/10/7/6	4/13/6/4
Local control (months) median	18.3	nr	nr
Survival (months) median; 95% CI	21.2; 10.5-28.7	28.8; 6.3-42.4	43.8; 12.4-

**Table 2:** HNSCC, postoperative radio- radiochemotherapy by PSMD1-score

**PSMD1-score**	**1 (n=26)**	**2 (n=27)**	**3 (n=39)**
Age (years) mean	63.4	60.8	60.2
Q1/Q2/Q3	58.5/64/70.2	52/60/70	51/61/68
Male (%)	88.4	81.4	73.6
Weight (kg) mean	64.9	68.8	67.7
Q1/Q2/Q3	57.8/64.5/73	56.8/68.8/77.5	57.8/68.7/77.6
Smoker (n)	7/26	17/26	17/39
Karnofski >= 70% (n)	19/19	19/20	30/30
Hemoglobin level (mg/dL) mean	12.7	12.7	12.0
Q1/Q2/Q3	12.0/12.6/13.6	11.1/13.1/14	11.2/11.9/12.9
Unknown primary		1 (3.7)	
Oral cavity n (%)	7 (26.9)	8 (29.6)	9 (23.0)
Oropharynx n (%)	9 (34.6)	10 (37.0)	16 ( 41.0)
Hypopharynx n (%)	5 (19.2)	2 (7.4)	8 (20.5)
Larynx n (%)	5 (19.2)	6 (22.2)	6 (15.3)
cT0 n (%)		1 (3.8)	3 (7.6)
cT1 n (%)	7 (26.9)	2 (7.6)	7 (17.9)
cT2 n (%)	8 (30.7)	8 (30.7)	11 (28.2)
cT3 n (%)	6 (23.0)	6 (23.0)	11 (28.2)
cT4 n (%)	5 (19.2)	9 (34.6)	7 (17.9)
cN0 n (%)	2 (7.6)	6 (22.2)	5 (12.8)
cN1 n (%)	5 (19.2)	4 (14.8)	14 (35.8)
cN2 n (%)	18 (69.2)	14 (51.8)	19 (48.6)
cN3 n (%)	1 (3.8)	3 (11.1)	1 (2.5)
G1 n (%)		1 (3.7)	1 (2.5)
G2 n (%)	12 (46.1)	15 (55.5)	21 (53.8)
G3 n (%)	14 (53.8)	11 (40.7)	17 (43.5)
EpoR (C20+) (%)	17 (65.3)	24 (88.8)	26 (66.6)
RT (Gy) mean	62.9	63.9	62.2
Q1/Q2/Q3	60/64/64	63/64/66	60/63/64
RT (days)	46.9	46.3	45.7
Q1/Q2/Q3	43/45/48	44/45/50	43.7/44.5/47.5
Treated in study A1/C3/D4 (n)	3/22/1	6/18/3	4/31/4
Local control (months) median	nr	nr	nr
Survival (months) median; 95% CI	48.2; 12.4-92.6	29.3; 13–74.1	42.8; 20.7-66.2

All trials were approved by the ethic committee of the University Hospital, Freiburg, Germany and done in accordance with the revised Declaration of Helsinki and good clinical practice guidelines. All patients provided written informed consent. The present study was additionally approved by the institutional review board of the University Hospital, Freiburg, Germany and the University of California, Los Angeles, USA.

Conventional or three-dimensional planning techniques were used for radiotherapy. The planning target volume (PTV) included the gross tumor volume (GTV) or tumor bed with a 1–2 cm safety margin and the regional lymph-node areas. 6 mega electron volt linear accelerators were used and standard dose and fractionation protocols (five fractions of 2.0 Gy or 1.8 Gy per week) were followed. A total dose of 60 Gy (allowable range 56–64 Gy) was prescribed to regions for R0 or R1 resected disease, and 70 Gy (allowable range 66–74 Gy) for primary definitive treatment or to macroscopically incompletely resected tumor (R2) and/or lymph nodes exceeding 2 cm. 50 Gy were administered to uninvolved nodal regions. The spinal cord was shielded after 30–36 Gy.

Follow-up was performed quarterly for the first two years, every six months for up to five years and continuously thereafter on a yearly basis. Locoregional tumor control and survival was assessed.

### Tissue microarrays

Tissue microarrays (TMAs) and immune-histochemical staining were used to analyze the expression of the proteasome subunit PSMD1 as previously described [21,25]. Briefly, TMA enables tumor tissue samples from different patients to be analyzed on the same histologic slide. A 2-mm needle was used to construct the array by extracting representative tumor tissue cores from each formalin-fixed, paraffin-embedded tissue blocks of HNSCC. TMA slides were counterstained with hematoxylin to visualize nuclei. PMSD1-expression analysis was performed by two pathologists who were unaware of the findings of the clinical data. A score of 1 was considered as ‘weak expression’, 2 was considered as ‘intermediate expression’, and a score of 3 was considered ‘strong expression’.

### Statistics

All experimental results are expressed as mean values. A *p-*value of ≤ 0.05 in a Student's *t*-test was considered to indicate statistically significant differences. The test was applied to normalized data to compensate for the variance of measurements between biologically independent replicates of the same experiments. CSC frequencies and *p* values were calculated using the Extreme Limiting Dilution Analysis (ELDA) software based on the algorithm defined by Hu and Smyth [31] (http://bioinf.wehi.edu.au/software/elda/). We confirmed that our data fits a single-hit linear model assumption by a likelihood ratio test to analyze goodness of fit.

The frequency of demographic and intervention parameters were descriptively determined in patients with different PSMD1-scores and locoregional tumor control and survival were assessed with Kaplan-Meier estimates within the different patient groups. Two-sided log-rank statistics were performed.

## Results

### HNSCC cells with low proteasome activity show increased self-renewal capacity

We had previously shown that breast cancer and glioma cells with low proteasome activity had a cancer stem cell phenotype, exhibiting increased self-renewal capacity and tumorigenicity [21]. Therefore we sought to explore if cells with intrinsically low proteasome activity could also be found in HNSCCs.

In order to assess proteasome activity in HNSCC lines we engineered UM-SCC4, UM-SCC6, UM-SCC12, UM-SCC-17B, Cal33 and FaDu cells to report the activity of this protease by accumulation of a fusion protein between the fluorescent protein ZsGreen and the C-terminal degron of murine ornithine decarboxylase (cODC). The latter directs the fusion protein to ubiquitin-independent degradation by the 26S proteasome. Therefore, cells with low proteasome activity accumulate the fluorescent fusion protein.

**Figure 1:**
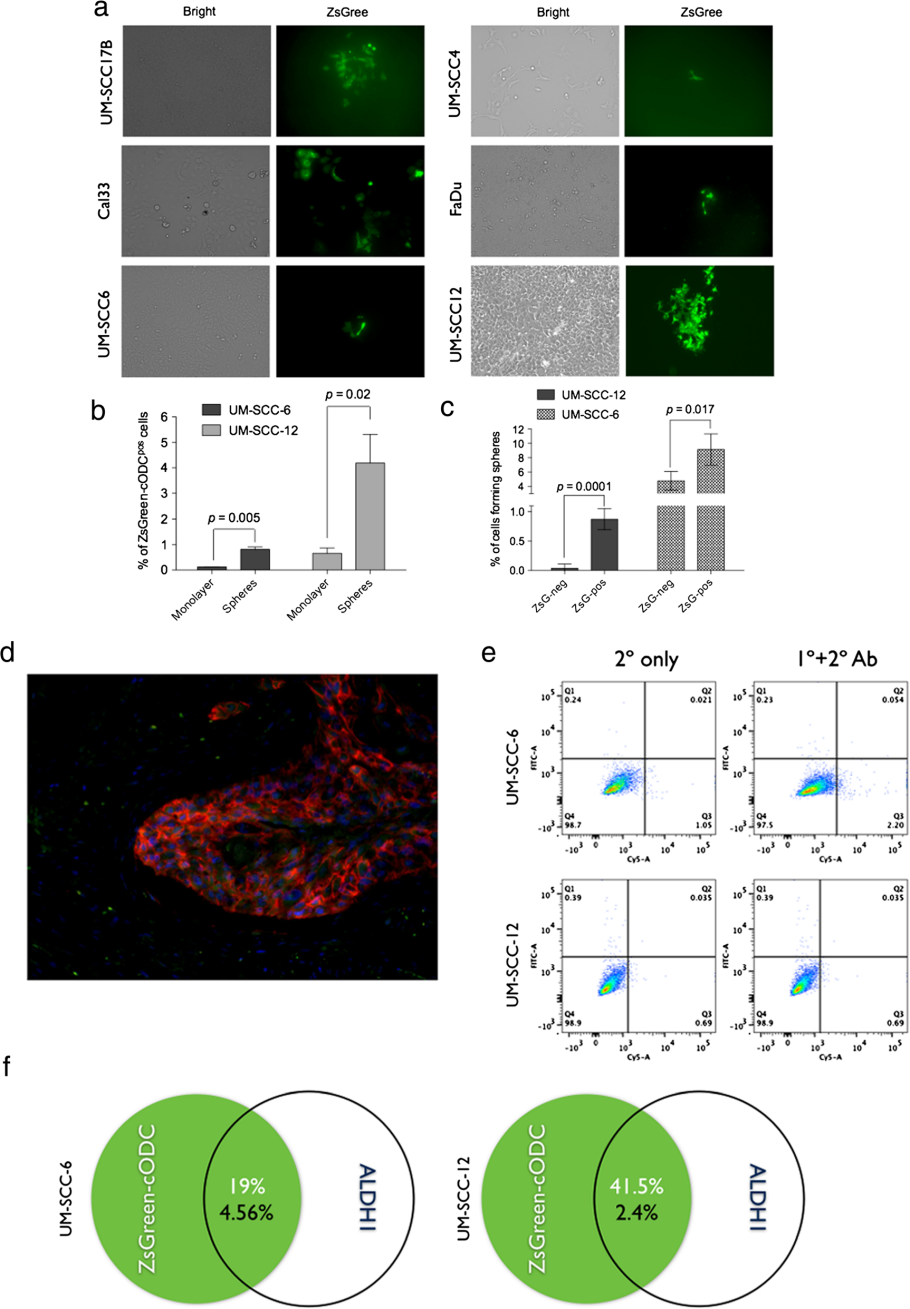
**HNSCC cell lines contain cell populations with low proteasome activity and higher sphere-forming capacity.** HNSCC cell lines were engineered to express the fusion protein ZsGreen and the c-terminal of the degron ornithine decarboxylase (cODC). **(a)** Cell lines were cultured in log-growth phase in DMEM, and representative bright field and green fluorescent pictures of monolayer cells are shown. **(b)** The percentage of ZsGreen-cODC^pos^ cells increases when HNSCC cells are cultured in serum-free media as tumorspheres. **(c)** Percentage of cells forming spheres from the ZsGreen-cODC^neg^ (ZsG-) and ZsGreen-cODC^pos^ (ZsG+) population after sorting by flow cytometry into 96-well plates. Means ± SD from four independent experiments are shown. **(d)** Representative CD44 staining of a HNSCC patient-derived tumor sample. Tumor cells show uniform membrane staining for CD44 (red). Nuclei are counterstained with DAPI (blue). **(e)** and **(f)** Flow cytometry analysis of ZsGreen accumulation (Y-axis) and ALDH1 expression (X-axis) in UM-SCC-6 and UM-SCC-12 cells). ZsGreen-cODC^pos^ cells with low proteasome activity are a subpopulation of ALDH1-expressing cells with 19% of ZsGreen-cODC^pos^ UM-SCC-6 cells and 41.5% of UM-SCC-12 cells positive for ALDH1.

When cells were kept as monolayer cultures, a low number of cells accumulated the fusion protein, thus indicating the presence of a small subpopulation of cells with intrinsically low proteasome activity (Figure 1a). When the UM-SCC6-ZsGreen-cODC and UM-SCC12-ZsGreen-cODC cells were grown in suspension as spheres in serum-free media supplemented with growth factors, the cultures were enriched in ZsGreen-cODC^pos^ cells (% UM-SCC6-ZsGreen-cODC^pos^ from monolayer: 0.12 ± 0.008;% UM-SCC6-ZsGreen-cODC^pos^ from spheres: 0.812 ± 0.19, *p* = 0.005, n = 4; (% UM-SCC12-ZsGreen-cODC^pos^ from monolayer: 0.655 ± 0.42;% UM-SCC12-ZsGreen-cODC^pos^ from spheres: 5.24 ± 0.97; *p* = 0.02, n = 4, two-sided Student’s t-test; Figure 1b). These growth conditions select for stem cells, while cells with limited proliferative potential die by anoikis. Furthermore, we sorted the UM-SCC6-ZsGreen-cODC and UM-SCC12-ZsGreen-cODC cells into ZsGreen-cODC^neg^ (high proteasome activity) and ZsGreen-cODC^pos^ (low proteasome activity) via FACS, and seeded these populations of cells into ultra-low adhesion plates in an *in vitro* limiting dilution assay (256 to 1 cells/well) under serum-free conditions and allowed for formation of tumor spheres. The sphere-forming capacity of these two subpopulations of cells differed in the two cell lines, however the ZsGreen-cODC^pos^ cells from both lines showed a significantly higher self-renewal capacity compared to the ZsGreen-cODC^neg^ cells (sphere forming capacity of UM-SCC6-ZsGreen-cODC^pos^ 9.15 ± 1.26%; UM-SCC6-ZsGreen-cODC^neg^ 4.77 ± 0.76% p = 0.041, n = 3; sphere forming capacity of UM-SCC12-ZsGreen-cODC^pos^ 0.88 ± 0.097%; UM-SCC12-ZsGreen-cODC^neg^ 0.038 ± 0.038% p = 0.0001, n = 4, two-sided Student’s t-test; Figure 1c). This data suggested that HNSCC are organized hierarchically or at least are heterogeneous with respect to their ability to self-renew.

In order to test if ZsGreen-cODC^pos^ cells in HNSCC overlap with cells positive for other established CSCs markers, HNSCC tumor sections were stained against CD44. CD44 caused a rather uniform membrane staining of the tumor cells (Figure 1d), which did not reflect the level of tumorigenicity seen in HNSCC xenografts studies.

The ZsGreen-cODC system cannot be used in combination with the Aldefluor assay, which uses a green-fluorescent substrate and therefore UM-SCC-6 and UM-SCC-12 cells were stained with an antibody against ALDH1 as described previously [20]. In both cell lines ZsGreen-cODC^pos^ cells with low proteasome activity were a subpopulation of ALDH1-expressing cells (Figure 1e and f).

To further confirm the tumor-initiating properties of the ZsGreen-cODC-positive population of cells we assessed the tumorigenicity of ZsGreen-cODC^pos^ and ZsGreen-cODC^neg^ cells *in vivo*. When UM-SCC12-cODC cells were injected into female nude mice, ZsGreen-cODC^pos^ showed a 20-fold higher tumorigenicity than ZsGreen-cODC^neg^ cells, thus suggesting that HNSCC cells with low proteasome activity are indeed highly enriched for CSCs (Table 3). The estimated frequencies of CSCs were 1 in 175,145 (CI: 410455 – 74737) in the ZsGreen-cODC^neg^ cell population and 1 in 48,942 (CI: 127,609 – 18,771) in the ZsGreencODC^pos^ cell population with ZsGreen-cODC^pos^ cells containing significantly more CSCs (p = 0.0315, Chi-Square test).

**Table 3:** ***In vivo *** **limiting dilution assay for UM-SCC12 cells**

**UM-SCC12-ZsGreen-cODC**		
**# of cells/innoculum**	**ZsG**^**neg**^	**ZsG**^**pos**^
100	0/10	0/10
1,000	0/12	2/10
10,000	1/10	3/8
100,000	4/10	2/4
1,000,000	4/4	Not performed

### Radiation treatment enriches for HNSCC cells with low proteasome activity

**Figure 2:**
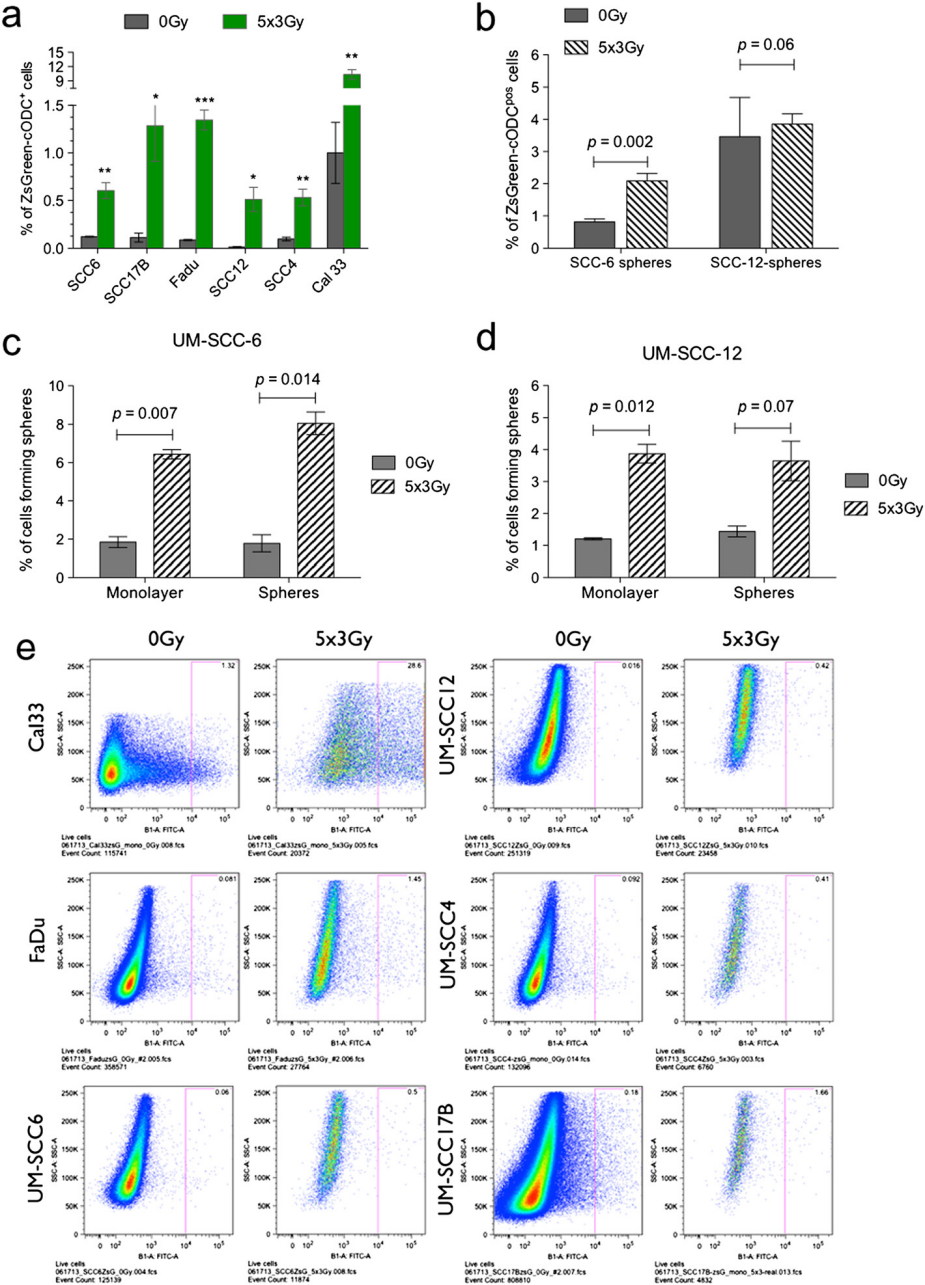
**Radiation enriches for cells with low proteasome activity and increases self-renewal capacity. (a)** HNSCC cells stably expressing the ZsGreen-cODC fusion protein were grown as monolayers cultures and treated with 5 daily fractions of 3 Gy. The number of ZsGreen-cODC^pos^ cells was assessed 72 hours after the last fraction of radiation using flow cytometry. Shown are mean percentages of ZsGreen-cODC^pos^ cells with standard deviation (SD). **(b)** Treatment of UM-SCC-6-ZsGreen-cODC and UM-SCC-12-ZsGreen-cODC sphere cultures were treated with 5 fractions of 3 Gy, also resulting in enrichment of ZsGreen-cODC^pos^ cells with low proteasome activity. This effect was more pronounced in radiosensitive [39] UM-SCC-6 cells than in radioresistant [40] UM-SCC12 cells. **(c and d)** 72 hours after the last fraction of radiation, cells were plated in 96-well plates at clonal densities to assess self-renewal capacity. Mean (± SD) percentages of cells forming a sphere with are shown. **(e)** Representative FACS analysis of HNSCC cell lines (monolayer) after treatment with 0 or 5×3 Gy.

Next, we tested if cells with intrinsically low proteasome activity would be intrinsically radioresistant. All the cell lines were seeded as monolayer cultures and treated with 5 daily fractions of 3 Gy. The number of ZsGreen-cODC^pos^ cells was assessed 72 hours after the last fraction of radiation, thus simulating a typical week of radiation treatment followed by a weekend gap. In all cell lines, fractionated radiation caused a significant increase in the percentage of ZsGreen-cODC^pos^ cells, suggesting that cells with low proteasome activity are indeed intrinsically radioresistant (Figure 2a and e). When the two different growth conditions were tested (monolayer vs. sphere media) with the UM-SCC-12-ZsGreen-cODC and UM-SCC-6-ZsGreen-cODC cells, the radiation-induced increase in ZsGreen-cODC^pos^ cells was seen regardless of the culture conditions chosen (Figure 2a and b).

### Radiation increases the self-renewal capacity of HNSCC cells

Next we assessed if radiation-induced increases in the number of ZsGreen-cODC^pos^ cells with low proteasome activity translated into increased self-renewal capacity. UM-SCC-6 and UM-SCC-12 were cultured as monolayers or tumorspheres and irradiated with 5 daily fractions of 3 Gy followed by a typical weekend gap of 72 hours. At this time, cells we seeded at clonal densities into ultra-low adhesions plates in sphere media. After 15 days, tumor spheres were counted. In both cell lines, irradiation caused a significant increase in self-renewal capacity for cells cultured as monolayers or tumorspheres (Monolayers: UM-SCC6, 0 Gy: 1.85 ± 0.28, 5×3 Gy: 6.434 ± 0.25, p = 0.007, n = 2; UM-SCC12: 0 Gy: 1.2 ± 0.03%, 5×3 Gy: 3.87 ± 0.29% p = 0.012, n = 2; Spheres: UM-SCC6, 0 Gy: 1.78 ± 0.45%, 5×3 Gy: 8.05 ± 0.59%, p = 0.014, n = 2; UM-SCC12, 0 Gy: 1.44 ± 0.17, 5×3 Gy: 3.65 ± 0.62% p = 0.075, n = 2, two-sided Student’s t-test; Figure 2c and d).

### Low proteasome subunit expression in HNSCC cells predicts treatment outcome

In order to test the clinical significance of cells with decreased proteasome activity in HNSCCs we used a tissue microarray that contained tumor samples of 82 HNSCC cases treated with primary definitive radiotherapy or radiochemotherapy. We previously described that lack of staining for the 19S proteasome regulatory subunit PSMD1 correlates with lack of 26S proteasome activity [21,25].

**Figure 3:**
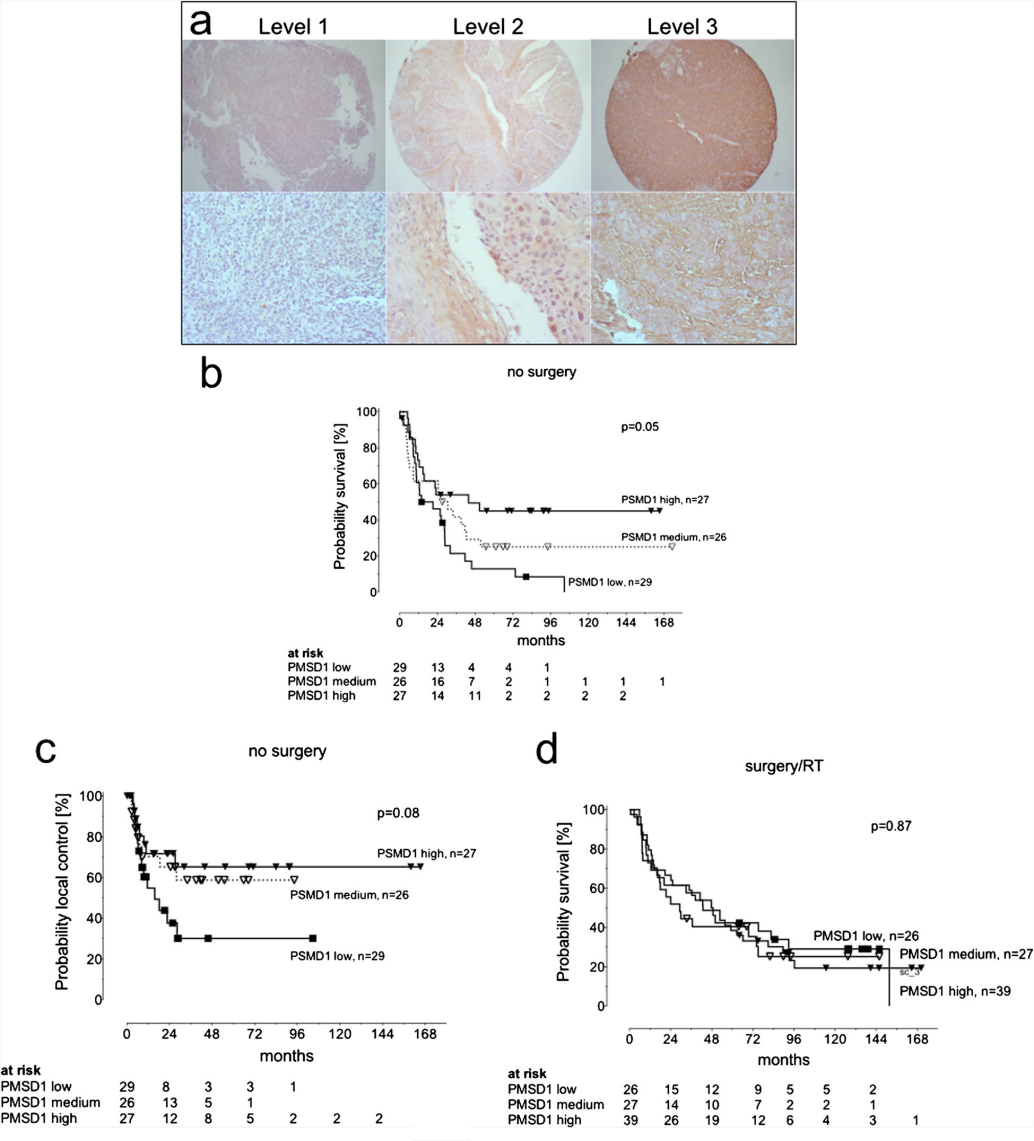
**Level of PSDM1 expression correlates with treatment outcome.** HNSCC tissue micro arrays were stained for PSMD1 to evaluate the expression of this proteasome subunit. The intensity of the staining was evaluated independently by two pathologists. **(a)** Pictures of representative staining for PSMD1 show the 3 different levels of staining. Overall survival **(b)** and loco-regional control **(c)** for patients receiving primary definitive radiotherapy. **(d)** Overall survival for patients receiving postoperative radiotherapy.

Figure 3a shows representative staining for levels 1–3 and Table 1 presents clinical data of patients by PSMD1-score. Characteristics of all three patient groups were quite similar. Possible imbalances in regards to tumor site, nodal involvement, erythropoietin receptor [32] or radiation may – if at all - favor patients with weak PSMD1-expression scores.

Kaplan-Meier estimates show that patients who underwent radiotherapy for macroscopic tumor and whose tumor cells exhibited weak or intermediate, as opposed to strong PSMD1 expression, had a decreased median overall survival probability (21.2 vs 28.8 vs 43.8 months, log-rank, p = 0.05) (Figure 3b). Comparably, a trend was observed for time to local tumor progression within the irradiated volume (p = 0.08, Figure 3c). This suggested that the number of cancer stem cells present during radiation treatment had an impact on treatment outcome.

In the case of patients in which the tumors could be resected successfully, expression of the proteasome subunit PSMD1 in cancer cells before surgery did not correlate with survival (Figure 3d). A description of these patients is given in Table 2.

## Discussion

We had previously reported that breast cancer [16] and glioma [21] cells with intrinsically low proteasome activity have a CSC phenotype. Similar results were reported for NSCLC [33] and pancreatic cancer [34]. Interestingly, in prostate cancer [35], breast cancer [16,36], and glioma [21,25] cells with low proteasome activity are radioresistant and patients with breast cancers [37] or gliomas [25] that express low levels of proteasome subunits have an unfavorable outcome. Recently, we reported that activation of the developmental Notch signaling pathway links the CSC phenotype with the proteasome. Musashi, a RNA binding protein crucial for maintaining Notch signaling, binds to the 3’-UTR of NF-YA mRNA, the master regulator of mammalian proteasome subunit expression, thereby down-regulating the proteasome in CSCs [38]. The intrinsic low proteasome activity in CSCs parallels with metabolic changes [25] and up-regulation of free radical scavenger systems, which ultimately cause radioresistance [17,36].

In the present study we show that HNSCC also contains a population of cells with low proteasome activity and decreased proteasome subunit expression and that these cells have a CSC phenotype defined by operational means. Like in breast cancer [16] or glioma [21], radiation enriches for these cells by selectively killing the more radiosensitive population with high proteasome activity and lower self-renewing capacity.

To our knowledge we show here for the first time that the number of cells with low proteasome activity present in HNSCCs inversely correlates with the overall survival of patients suffering from HNSCC. It is unlikely that design, conduct or patient selection contributed to this finding. The clinical samples were derived from prospective trials where data collection, validation, and processing followed good clinical practice; the adherence to study protocols was ascertained and a continuous follow-up for nine years sufficiently substantiates our observation. Although the sample size is limited, essential methodological pitfalls seem not to confound our observations. Baseline and treatment characteristics are reasonably balanced, immune-histochemical processing is standardized by TMA-methodology, adequate controls were used, and two unbiased, independent researchers, blinded for all clinical parameters, performed the evaluation.

PSMD1 expression seemed to also affect the locoregional cancer control probability of our patients undergoing primary definitive radiotherapy and we propose that treatment outcome was predominantly driven by an impaired treatment efficacy based on an increased number of therapy resistant CSCs. Furthermore, our *in vitro* data suggested that radiation enriches for CSCs and increases self-renewal capacity of HNSCC cell populations. Finally, the number of CSCs in patients in which the tumor could be resected was not related to the prognosis (Table 2 and Figure 3), thus supporting the relevance of the total number of CSCs for overall survival. One can speculate that the very low number of CSCs in subclinical disease in those patients will most likely be controlled by standard radiotherapy regimens.

## Conclusions

We conclude that HNSCCs contain subpopulations of cells with CSC features, which can be identified by lack of proteasome activity and low proteasome subunit PSMD1-expression. HNSCC CSCs are of clinical relevance because they affect tumor control and survival. Thus, PSMD1-testing could be useful in identifying patients with HNSCC at risk for relapse.

## Competing interests

The authors have declared that no conflict of interest exists.

## Authors’ contributions

CL performed the *in vitro* and *in vivo* experiments, EV performed the *in vitro* and *in vivo* experiments and wrote the manuscript, SB and CL scored the tissue micro arrays, PM and MW were responsible for the TMA assembly and staining, MH collected and analyzed the clinical data, FP conceived of the study, designed the experiments, analyzed the data and wrote the manuscript. All authors read and approved the final version of the manuscript.

## Pre-publication history

The pre-publication history for this paper can be accessed here:

http://www.biomedcentral.com/1471-2407/14/152/prepub
